# A randomized pilot phase I study of modified carcinoembryonic antigen (CEA) peptide (CAP1-6D)/montanide/GM-CSF-vaccine in patients with pancreatic adenocarcinoma

**DOI:** 10.1186/2051-1426-1-8

**Published:** 2013-06-27

**Authors:** Daniel M Geynisman, Yuanyuan Zha, Rangesh Kunnavakkam, Mebea Aklilu, Daniel VT Catenacci, Blase N Polite, Cara Rosenbaum, Azadeh Namakydoust, Theodore Karrison, Thomas F Gajewski, Hedy L Kindler

**Affiliations:** 1 Section of Hematology/Oncology, University of Chicago, Chicago, IL, USA; 2 University of Chicago Cancer Research Center, Chicago, IL, USA; 3 Department of Health Studies, University of Chicago, Chicago, IL, USA; 4 Section of Hematology/Oncology, Department of Medicine, University of Chicago, 5841 South Maryland Avenue, MC 2115, Chicago, IL 60637, USA

**Keywords:** Pancreatic Cancer, Vaccine, Immunization, CEA

## Abstract

**Background:**

CEA is expressed in >90% of pancreatic cancers (PC) and may be an appropriate immunotherapy target. CEA is poorly immunogenic due to immune tolerance; CAP1-6D, an altered peptide ligand can help bypass tolerance. We conducted a pilot randomized phase I trial in PC patients to determine the peptide dose required to induce an optimal CD8^+^ T cell response.

**Methods:**

Patients with a PS 0-1, HLA-A2+ and CEA-expressing, previously-treated PC were randomized to receive 10 μg (arm A), 100 μg (arm B) or 1000 μg (arm C) of CEA peptide emulsified in Montanide and GM-CSF, given every 2 weeks until disease progression.

**Results:**

Sixty-six patients were screened and 19 enrolled of whom 14 received at least 3 doses of the vaccine and thus evaluated for the primary immunologic endpoint. A median of 4 cycles (range 1-81) was delivered. Median and mean peak IFN-γ T cell response by ELISPOT (spots per 10^4^ CD8^+^ cells, Arm A/B/C) was 11/52/271 (A vs. C, p = 0.028) for medians and 37/148/248 (A vs. C, p = 0.032) for means. T cell responses developed or increased in 20%/60%/100% of pts in Arms A/B/C. Seven of the 19 patients remain alive at a minimum 32 months from trial initiation, including three with unresectable disease.

**Conclusions:**

The T cell response in this randomized phase I trial was dose-dependent with the 1 mg CEA peptide dose eliciting the most robust T cell responses. A signal of clinical benefit was observed and no significant toxicity was noted. Further evaluation of 1 mg CEA peptide with stronger adjuvants, and/or combined with agents to overcome immune inhibitory pathways, may be warranted in PC pts.

**Trial registration:**

ClinicalTrials.gov NCT00203892

## Background

Pancreatic cancer (PC) is a devastating disease. With an estimated annual incidence of 43,920 in the United States in 2012, it is the fourth leading cause of cancer mortality with 37,390 deaths annually [1]. Currently, the only possibility of cure is through surgical resection, but only approximately 15%–20% of patients are eligible candidates at diagnosis and even with surgery the 5-year overall survival in multiple series is 10%-30% [2]. In those with locally advanced or unresectable disease (~30%–40% of patients) the median overall survival (OS) has been between 8 and 14 months. Patients with metastatic disease (~40%) had a historical median OS of approximately 6 months, although recently substantial progress has been made in chemotherapy options for these patients [3].

Due to the inherent chemotherapy- and radiation-resistance of pancreatic cancer cells, immunotherapy has been explored as a treatment modality in PC patients since the 1990s with a recent review focused on this topic [4]. Multiple vaccination platforms have been developed and tested in advanced solid malignancies including PC, both in metastatic and adjuvant settings. Most of the vaccination techniques have focused on promoting a tumor antigen-specific T cell response using either vaccination or adaptive transfer of tumor-specific T cells in order to increase immune-mediated tumor inhibition [5]. Identification of specific tumor-associated antigens (TAAs) in PC has led to numerous trials of vaccination with these peptides as this approach is safe and relatively simple [6-13]. The goal of this technique is to elicit a CD8^+^ cytolytic T lymphocyte (CTL) response that is specific for the TAA and subsequent killing of tumor cells harboring that antigen by those CTLs.

An example of a TAA, carcinoembryonic antigen (CEA), is a 180-kDa immunoglobulin-like molecule that is expressed on the cell surface, is overexpressed in over 90% of pancreatic adenocarcinoma and functions in cellular adhesion [12,14,15]. CEA as a self-protein has been shown to be poorly immunogenic and thus modifications have been made to enable better binding to the MHC-I complex. CAP-1 (YLSGANLNL), an epitope of CEA, was further modified to CAP1-6D (an Asn to Asp substitution in the CEA sequence; YLSGADLNL), and is an enhanced agonist peptide binding to HLA-A2. Via a change in the interaction with the T cell receptor it produces a more potent CTL response and T cells generated via this approach have been shown to be cross-reactive with wildtype CAP1 and to recognize CEA^+^ HLA-A2^+^ tumor cells [16,17].

A number of trials have employed the CEA TAA in various vaccination platforms for patients with CEA-expressing tumors. The early trials showed that the approach was safe in various platforms and with GM-CSF as an adjuvant [18-21]. In the trials which incorporated CEA as an antigen, only one to our knowledge was conducted and reported exclusively in PC patients [22]. In that phase I study, a poxvirus targeting CEA and MUC-1 along with B7.1, ICAM-1 and LFA-3 (TRICOM) (PANVAC-V) was administered followed by booster vaccinations using PANVAC-F (fowlpox virus with same antigens) as well as GM-CSF in ten advanced PC patients. They noted an antibody response in all 10 patients and antigen-specific T cell responses in 5 out of 8 evaluable subjects. A significant increase in OS was observed in those who mounted an anti-CEA and/or MUC-1 response (15.1 m vs. 3.9 m; P = 0.002). Several other studies used CEA vaccination alone or with other epitopes such as MUC-1, with or without radiotherapy and with various platforms, but most patients in those trials did not have PC [23-27]. In general, the vaccines were all safe, elicited an immunologic response in a significant number of patients, and led occasionally to sustained clinical responses.

Given the above considerations, we believed it was desirable to explore further CEA-based vaccination in PC patients. To eliminate the necessity of obtaining patient-specific DCs, we utilized tumor antigen peptide emulsification in Montanide adjuvant along with GM-CSF, which has been suggested to stimulate DC differentiation and improve DC recruitment [28,29]. Because the appropriate dose of a CEA-based vaccine to elicit the most robust response was unknown, we designed a randomized phase I study to determine the most appropriate peptide dose. This approach would enable us to ascertain whether an immune response could be elicited, and could establish the most effective dose of the CEA peptide within the vaccine formulation to use in further trials. We report the results of this randomized phase I pilot trial of a CAP1-6D/Montanide/GM-CSF-vaccine in PC patients (ClinicalTrial.gov ID: NCT00203892).

## Methods

### Trial design

The study was designed as a randomized pilot trial with the primary endpoint to determine the dose of modified carcinoembryonic antigen (CEA) peptide (CAP1-6D)/Montanide/GM-CSF-vaccine required to induce an optimal CD8^+^ T cell response and to determine whether this response can be assessed by ELISPOT. Defining the dose limiting toxicities (DLTs), progression free survival and median overall survival were secondary endpoints. The laboratory objective was to determine whether this immunization elicits a specific T cell response as assessed by IFN-γ ELISPOT, against both the CAP1-6D and the native peptide. The study was approved by the University of Chicago Institutional Review Board and conducted at the University of Chicago. All patients were required to give written informed consent in accordance with federal, state, and institutional guidelines.

### Eligibility

Patients were required to have an Eastern Cooperative Oncology Group performance status of 0 or 1 and adequate hematologic, hepatic, and renal function. Although originally designed to include only those treated definitively with no evidence of disease (NED) or those with locally advanced PC, the inclusion was later expanded to allow for those with metastatic PC in order to improve accrual. Eligible patients must have expressed HLA-A2 and have histologically or cytologically confirmed adenocarcinoma of the pancreas that expresses CEA either by IHC or serology. Life expectancy of over 3 months, no systemic treatment within 28 days of trial initiation (6 weeks for nitrosoureas or mitomycin C), and age of 18 or older were also required. Exclusion criteria included previous CEA vaccine; history of allergic reaction to compounds of similar chemical or biologic composition to CEA, Montanide ISA-51 or GM-CSF; known autoimmune disorders; conditions of immunosupression such as HIV or treatment with immunosuppressive drugs; pregnancy or breast-feeding; currently active second malignancy; and uncontrolled intercurrent illness.

### Vaccine formulation and treatment plan

The vaccine contained the modified CEA peptide (CAP1-6D; YLSGADLNL; Multiple Peptide Systems, San Diego, CA) together with Montanide ISA-51 (Seppic, Inc.) as an adjuvant, and sargramostim (GM-CSF; purchased commercially) 250 μg. Vaccine emulsions were prepared in the University of Chicago cGMP facility. Briefly, the appropriate dose of CEA peptide was thawed, combined with 0.9 ml of sterile saline and mixed with GM-CSF and Montanide ISA-51 using the two-syringe method to make an emulsion. To verify creation of an emulsion, a drop was placed in a dish of sterile water and if the drop did not disperse, the emulsion was considered to be a success.

Patients were seen for a baseline evaluation which included a history and physical, CT/MRI, blood chemistries, complete blood count with differential, and HLA typing. Confirmation of CEA expression was established either by IHC on the original tumor or by elevated serum CEA levels. Only subjects positive for HLA-A2 and with evidence for CEA expression continued on the study. A cycle was defined as 14 days and vaccine was administered on Day 1 of each cycle until progressive disease or dose-limiting toxicity for a maximum of 24 cycles with delays of greater than 28 days leading to study withdrawal. The vaccine administration site was the proximal thigh with each subsequent administration in the same approximate location. Following the initial administration, patients were seen prior to each vaccine administration with ELISPOT and CA19-9 performed every 4 weeks for the first 8 cycles and a CT/MRI every 8 weeks. After the eighth cycle, CT/MRI and CA 19-9 was repeated every 8 weeks thereafter for one year until disease progression or DLT with ELISPOT performed at the time of disease progression. After one year, CT scans were done every 16 weeks for one year, followed by every 6 months for one year and then annually until progression of disease.

### Response criteria and toxicity

Clinical response was evaluated using the Response Evaluation Criteria in Solid Tumors v1.0 [30]. Dose-limiting toxicity was defined as Grade 2 or greater hemorrhage or allergic reaction; any other Grade 3 or greater toxicity or clinical evidence of autoimmune disease. All adverse events were graded according to the CTCAE v2.0.

### Collection of peripheral blood mononuclear cells (PBMCs) and preparation of CD8^+^ T cells

Heparinized blood was drawn before treatment, monthly during the vaccination period, and at the end of study. Samples were collected prior to a given treatment administration. PBMCs were isolated using Lymphoprep gradient centrifugation and cryopreserved for immune assays. CEA-specific CD8^+^ CTL responses were tested by IFN-γ ELISPOT, against both the modified CEA (CAP1-6D) and the wild-type peptide. Poor CEA-specific CD8^+^ CTL responses were detected by a direct ex vivo assay, so a short in vitro expansion was performed. Briefly, PBMCs were thawed and washed twice with PBS. CD8^+^ T cells were isolated using anti-CD8 micro beads (Miltenyi Biotech). The non-CD8^+^ population was pulsed with modified CEA peptide (50 μM) at the presence of β2-microglobulin for 1 hour at 37˚C. The cells were then washed twice with AIM-V media and irradiated at 3000 rad. Purified CD8^+^ T cells were stimulated with irradiated peptide-pulsed CD8^-^ cells along with IL-2 (10 U/ml) for 5 days. On day 5, the CD8^+^ T cells were collected and re-stimulated with freshly prepared irradiated peptide-pulsed CD8^-^ cells and IL-2 (10 U/ml) for another 5 days. On day 10, the expanded CD8^+^ T cells were collected, counted, and re-stimulated with peptide-loaded T2 cells for IFN-γ ELISPOT analysis.

### ELISPOT assay

Briefly, 96 well multiscreen filter plates were prepared by coating overnight with anti-INF-γ mAb (10 μg/ml), washing 3× with PBS, and blocking 1 hour with AIM-V medium containing 10% human AB serum. Expanded CD8^+^ T cells (10,000/well) were added along with T2 cells (50,000/well) previously loaded with wild type CEA peptide (CAP1), modified CEA peptide (CAP1-6D) and a G250 negative control peptide (50 μM each). Following a 20 hour culture, wells were washed 3 times with ELISPOT wash buffer, incubated 2 hours with a biotinylated anti-IFN-γ secondary Ab, washed 3 times, incubated 1 hour with streptavidin-conjugated AP, washed, and incubated with AP substrate. Excess substrate was removed by rinsing with tap water. Plates were captured and counted using a CTL-ImmunoSpot S6 Core Analyzer from Cellular Technology Ltd (Cleveland, OH). Stimulation with PMA + Ionomycin was used as a positive control for the integrity of the T cell samples. All samples were analyzed in triplicate, and the mean response to the G250 negative control was subtracted from each sample. A positive ELISPOT response for a patient was defined as a minimum increase from baseline in a CTL ELISPOT assay by 50 CEA spots/1×10^4^ CD8^+^ T cells and a 20% increase over baseline: [(peak ELISPOT-baseline ELISPOT)/baseline ELISPOT] × 100 ≥ 20% and a peak ELISPOT ≥ 50 spots.

### Statistical considerations

Patients were randomized into one of three dose levels. A sample size of 15 evaluable patients was planned to obtain sufficient T cell response data in each cohort. The sample size was based on a recombinant avipox vaccine study by von Mehren et al. in patients with recurrent CEA-expressing adenocarcinomas [24]. Acceptable toxicity was defined as a DLT observed in no more than 20% of patients at a given dose.

To be included in the analysis a patient must have had a baseline ELISPOT and then at least 3 cycles of the vaccine with an additional ELISPOT done after the 3^rd^ cycle. The maximum T cell frequency achieved was used in the calculation. Descriptive statistics were used to describe the characteristics of the study population.

Data was analyzed for overall survival and progression free survival using the Kaplan-Meier estimates from the start of treatment and comparison between the treatment arms using the log-rank test. Elispot responses were compared using Mann–Whitney Rank Sum Test with a *P* value based on a two-tailed test and P < 0.05 considered statistically significant. Univariate Cox regression models examining CA19-9 levels, ELISPOT and stage (metastatic vs. non-metastatic) were done for PFS and OS.

## Results

### Patient characteristics

**Figure 1:**
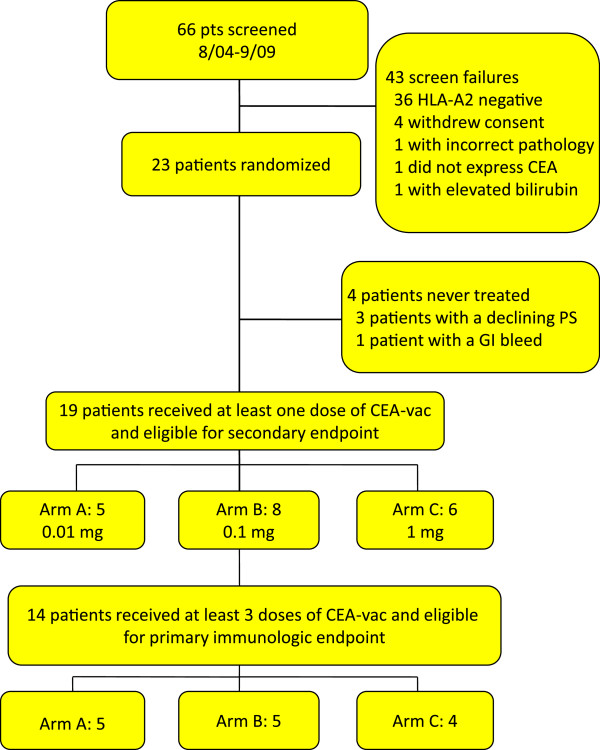
**Study Enrollment and Treatment.** CONSORT Diagram.

Sixty-six patients were screened, twenty-three enrolled, and nineteen received at least a single dose of the vaccine (Figure 1). Patients were randomized to receive 10 μg (arm A), 100 μg (arm B) or 1000 μg (arm C) of peptide in the vaccine preparation. Forty-three subjects were screen failures: 36 due to lack of HLA-A2 expression, four for withdrawing of consent, one for incorrect pathology, one for lack of CEA expression by IHC, and one for an elevated bilirubin. Nineteen patients received at least one dose of the vaccine; three patients experienced a declining performance status and one had a gastrointestinal bleed prior to administration of any vaccine. Fourteen patients received at least three doses of the vaccine and were eligible for the primary immunologic endpoint: 5 in arm A, 5 in arm B and 4 in arm C. The overall and per-group patient characteristics are listed in Table 1. Sixty-eight percent of patients were female. The median age was 60 (range 27–86) and 53% had an ECOG performance status 0. Three-quarters had metastatic disease, with all but one of the remaining patients having no evidence of disease at the time of enrollment.

**Table 1:** Overall and per-treatment-group patient baseline characteristics

**Baseline characteristics**	**Overall no. of patients (%)**	**Arm A (%)**	**Arm B (%)**	**Arm C (%)**
Total	19	5	8	6
Age				
Median	60	51	57.5	61.5
Range	27–86	27–71	50–86	47–77
Sex				
Male	6 (32)	1 (20)	4 (50)	1 (17)
Female	13 (68)	4 (80)	4 (50)	5 (83)
ECOG Performance Status				
0	10	2	4	4
1	9	3	4	2
Stage at Trial Entry				
No evidence of disease	4 (21)	2 (40)	0 (0)	2 (33)
Locally advanced	1 (5)	0 (0)	0 (0)	1 (17)
Metastatic*	14 (74)	3 (60)	8 (100)	3 (50)
Prior Therapy				
Chemotherapy alone	5	1	3	1
Chemotherapy/RT alone	2	0	1	1
Surgery total	12	4	4	4
Surgery followed by chemotherapy	7	3	2	2
	5	1	2	2
Surgery followed by chemo/RT				
Prior surgical resection				
Yes	12 (63)	4 (80)	4 (50)	4 (67)
No	7 (37)	1 (20)	4 (50)	2 (33)
Serum CEA at trial initiation (ng/mL)				
Median	4.8	5.3	4.65	7.95
Range	1–246.7	1.1–34.2	2.1–246.7	1–18.1
Serum CA-19-9 at trial initiation (ng/mL)				
Median	532	22	1067	27
Range	3–96,900	3–2,710	3–96,900	3–30,300

Abbreviation: Chemo/RT = chemotherapy in combination with radiation;

* All received previous chemotherapy.

A total of 256 cycles were delivered (median 4, mean, 13.5, range 1–81). The initial protocol did not specify a maximum number of cycles and this was amended after the first patient received 81 cycles, at which time the number of cycles was limited to 24. No dose reductions were allowed nor did any occur.

### Immunologic response

**Figure 2:**
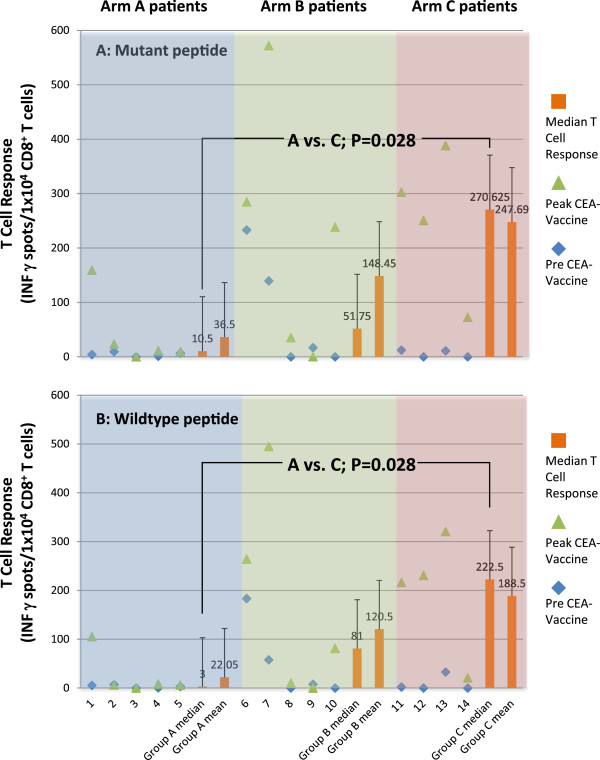
**Baseline and Peak ELISPOT results for 14 evaluable patients and median and mean *CAP1-6D *(Panel A) *and wild-type *(Panel B) T Cell response per dose level.** Baseline (blue diamond) and peak (green triangle) T cell responses by ELISPOT for each of the 14 evaluable patients. Median and mean T cell response by ELISPOT (spots per 10^4^ CD8^+^ cells) measured after at least 3 cycles is indicated by the orange bars per each arm. For ***CAP1-6D*** (Panel **A**) median/mean Arm A (0.01 mg) response = 10.5/36.5; median/mean Arm B (0.1 mg) response = 51.75/148.45; median/mean Arm C (1 mg) response = 270.63/247.69. *P* = 0.028 as measured by Mann–Whitney Rank Sum Test comparing medians. For ***wild-type*** (Panel **B**) median/mean Arm A (0.01 mg) response = 3/22.05; median/mean Arm B (0.1 mg) response = 81/120.5; median/mean Arm C (1 mg) response = 222.5/188.5. *P* = 0.028 as measured by Mann–Whitney Rank Sum Test comparing medians.

**Figure 3:**
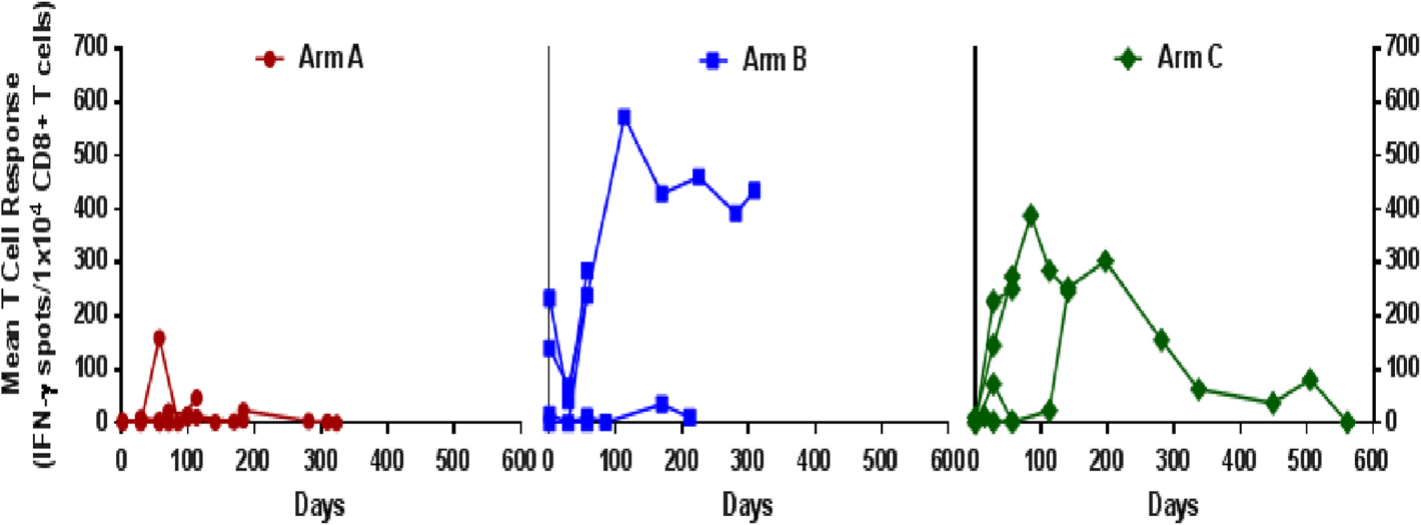
**ELISPOT kinetics of *in vitro *primed CAP-1 peptide specific-CD8+ T cell responses.** T cell responses for all 14 patients at various time points on the trial are presented and stratified by treatment arm. Day 1 is first day of vaccination.

A positive CD8^+^ T cell response to CAP1-6D developed in 20% of patients in Arm A, 60% of patients in Arm B, and 100% of patients in Arm C. Five patients were not able to be assessed immunologically due to either less than 3 cycles of the vaccine administered (one patient) or for incomplete sample collection due to early disease progression (4 patients). The mean/median CAP1-6D CD8^+^ T cell response by ELISPOT in the 14 evaluable patients (spots per 10^4^ CD8^+^ cells) was 36.5/10.5 in Arm A, 148.45/51.75 in Arm B and 247.69/270.625 in Arm C (Figure 2). A statistically significant difference in median (P = 0.028) and mean (P = 0.032) T cell response against the immunizing mutant peptide was observed between arm A and C. T cell responses to the wild-type CEA peptide followed a similar pattern and also were statistically significantly different between arm A and C (P = 0.028) (Figure 2). The kinetics of the magnitude of the T cell responses were tracked over time for all 14 patients stratified by treatment arm (Figure 3). In general, the peak immune response appeared to occur within the first 100 days and diminished thereafter. In the two of the three patients receiving the vaccine for over 4 months the response was still sustained and remained detectable.

### Response and survival

Individual clinical responses for all 19 patients are listed in Table 2. Seven patients (37%) had stable disease (SD) as their best response and all remain alive today. Among the 14 immunologically evaluable patients, eight had a positive ELISPOT response of which 4 had progressive disease (PD), 3 SD and 1 a complete response (CR) as their best response. Among the 6 patients failing to exhibit positive ELISPOT response, 2 had PD and 4 SD as their best response. There was one CR in a patient with locally advanced, unresectable PC. This patient underwent chemotherapy and radiation and then received 81 doses of the vaccine, randomized to Arm C. She developed a strong ELISPOT response and remains alive and disease free at over 75 months. One patient with metastatic PC in Arm B had SD for 11 months (22 cycles of vaccine administered), a strong CTL response and is alive at over 43 months. Another patient with metastatic PC in Arm C had SD for 6 months, a strong CTL response and is alive at over 48 months. Of the 5 patients that were not able to be evaluated for immunologic response, 3 were in Arm B and 2 in Arm C (Table 2). All 5 had PD within less than 2 months and all died shortly thereafter. Eleven patients had progressive disease (PD) as their best response at or prior to their first scheduled CT evaluation at 8 weeks.

**Table 2:** Response and survival in all 19 patients

**Arm**	**Stage**	**ELISPOT response (Y/N/NE)**	**Best response**	**No. of cycles completed**	**Time on trial (months)**	**Months alive from trial start**
A	M	Y	SD	8	3.7	9.4
A	M	N	PD	4	1.8	15.9
A	M	N	SD	24	11.1	47.2*
A	NED	N	SD	24	12.4	32.4*
A	NED	N	SD	24	12.2	31.9*
B	M	Y	PD	4	1.9	3.1
B	M	Y	SD	23	12	43.2*
B	M	Y	PD	3	1.4	3.6
B	M	N	SD	22	10	?*
B	M	N	PD	4	1.8	2.2
B	M	NE	PD	1	0.3	0.4
B	M	NE	PD	4	1.2	1.5
B	M	NE	PD	4	1.8	3.0
C	M	Y	PD	4	1.9	11.3
C	LA	Y	CR	81	39.3	75.4*
C	M	Y	PD	4	1.8	11.0
C	M	Y	SD	12	6.2	48.5*
C	M	NE	PD	3	1.2	2.0
C	M	NE	PD	3	1.4	2.6

Abbreviations: NED = No evidence of disease; M = metastatic; LA = locally advanced; Y = Yes; N = No; NE = not evaluable; * = currently alive; ? = patient is known to be alive but had withdrawn consent; Data as of May 22, 2012.

The median OS for the entire cohort of 19 patients was 334 days with a median PFS of 56 days. Overall, 7/19 patients (37%) were alive at a minimum 32 months from trial initiation and of the 17 patients with LA or metastatic disease, five (29%) remain alive. The study was not powered to detect differences in clinical outcome between the three cohorts.

### Toxicity

All 19 patients were evaluable for toxicity. No grade 4 and 5 toxicities were observed; grade 1-3 toxicities are summarized in Table 3. No discontinuation of treatment occurred due to toxicity and no grade 2 or greater hemorrhage or allergic reaction occurred; no autoimmune disorders were noted. The most common toxicities were grade 1-2 skin reactions to the vaccine injection. These did not develop differentially in responders versus non-responders and did not correlate with ELISPOT results. Otherwise common side effects of pain, fatigue, anorexia, nausea/vomiting, diarrhea and constipation were felt to be due to primary disease and not the study vaccine.

**Table 3:** Grade 2 or greater toxicities associated with vaccine

	**Grade 1**	**Grade 2**	**Grade 3**	**Grade 4**
**Toxicity**	**No. (%)**	**No. (%)**	**No. (%)**	**No. (%)**
Injection-site reaction	11 (53)	1 (5)	0 (0)	0 (0)
Erythema	9 (47)			
Induration	6 (32)	1 (5)		
Pain	3 (16)			
Ecchymosis	2 (11)			
Discoloration	1 (5)			
Edema	1 (5)			
Rash	1 (5)			
Reaction NOS	1 (5)			
Toxicity unrelated to injection site	10 (53)	3 (16)	4 (21)	0 (0)
Pain	10 (53)	3 (16)	2 (11)	
Fatigue	10 (53)	1 (5)	4 (21)	
Anorexia	4 (21)	0 (0)	2 (11)	
Nausea/Vomiting	8 (42)	0 (0)	1 (5)	
Constipation	6 (32)	2 (11)	1 (5)	
Diarrhea	3 (16)	1 (5)	1 (5)	

## Discussion

The diagnosis of pancreatic cancer is associated with approximately a 5-year survival rate of 6% [1]. Innovative therapies are thus desperately needed. Immunotherapy via vaccination has been one approach investigated over the last decade. In pancreatic cancer, several trials have shown that use of a TAA such as MUC-1 in combination with CEA or K-Ras can elicit an immunologic response and possibly improve outcomes in those mounting such a response. To our knowledge, no other trial reported has used CEA as the exclusive TAA vaccination platform in a strictly PC population and the optimal dose of such a vaccine was unknown. The primary objective of this study was to determine the optimal dose of the carcinoembryonic antigen (CEA) peptide (CAP1-6D)/Montanide/GM-CSF-vaccine amongst the three doses administered to induce a maximal CTL response in patients with pancreatic cancer.

Based on our data of 14 immunologically assessable patients, the 1 mg dose of peptide in the vaccine emulsion led to the maximal T cell response. A dose response between the vaccine peptide dose and an induced T cell response was observed, with 100% of patients in the 1 mg arm who were eligible for immunologic evaluation demonstrating a CD8^+^ T cell response by IFN-γ ELISPOT. The median OS and PFS were 334 and 56 days, respectively, and the vaccination was safe and well tolerated. We believe that the randomized phase I trial design proved to be a useful tool, as it allowed determination of the optimal dose of peptide for induction of an antigen-specific CD8^+^ T cell response in this patient population.

Our study does have a number of limitations that highlight some important issues in cancer vaccine development. First, although our sample size was sufficient to answer the primary scientific objective, it was not large enough to have meaningful comparisons of clinical outcomes among the three groups. Also, no obvious correlation between a positive ELISPOT response and clinical outcome was noted, but this is not unexpected due to known resistance mechanisms in the tumor microenvironment and the study’s small sample size. Second, due to the inherent need for HLA-A2 restriction that is imposed by using a peptide antigen, many potential subjects were screen failures. In that regard, vaccination platforms that do not restrict based on HLA status, such as those based on whole protein or full length cDNAs incorporated into suitable vectors, do have advantages. Third, CEA is not necessarily utilized by pancreatic cancer cells as part of the malignant process, and it could be argued that gene products contributing to cancer cell growth or survival may be more desirable to target. Finally, in order to enable sufficient accrual, we did not have a homogenous patient population, allowing the inclusion of metastatic, resected, and locally advanced patients, thus not permitting definitive conclusions to be made about any one group.

Of note, two patients in group A were enrolled into the study with NED and failed to develop an immune response. We do not have alternate indices of general immune competence for them to try to further explain their anergic state, but the stimulation with PMA + Ionomycin was positive. As noted above and presented in Figure 3, the kinetics of the T cell response suggest that if a response occurred, it happened early with the maximum response occurring within the first 100 days. For those on trial for over 4 months, the response does appear to wane over time, although was sustained at over 300 days for two patients. Also, in regard to vaccine-site delayed-type hypersensitivity as evidenced by skin reactions we did not notice a particular pattern to correlate with ELISPOT results. Out of the 11 patients with a reaction, some developed it without ever mounting a positive ELISPOT results, some at the time of their peak and some at a different point in time.

Three phase III trials of PC vaccines have been conducted previously, all of which did not meet their primary objective [31-33]. Over 20 vaccine immunotherapy clinical trials are currently ongoing in various stages of completion (http://clinicaltrials.gov) examining varying iterations of the above approaches for cancers including but not limited to PC, including novel peptides (e.g., mesothelin, VEGFR1/VEGFR2 and survivin), DNA-based vaccines, and combinations of vaccines with chemotherapy. Most results in PC thus far have been underwhelming and the field is still searching for the optimal vaccine approach, clinical context, and predictive biomarkers of clinical benefit. Nonetheless, the favorable clinical outcome observed in a subset of patients treated in our current CEA vaccine trial motivates continued investigations of immunotherapeutic strategies in this disease.

Our vaccination approach utilized the adjuvant Montanide ISA-51 along with the cytokine GM-CSF included in the emulsion. It is not clear that this is an optimal vaccine adjuvant for peptide vaccines, and further improvements in the vaccine formulation are conceivable. After this trial was initiated, Slingluff and colleagues reported that the inclusion of GM-CSF with Montanide may result in diminished peptide-induced T cell responses in a melanoma vaccine study [34]. The TLR agonists CpG 7909 and polyIC:LC have recently been explored and should continue to be investigated as a possible adjuvant [35]. The MAGE-3 protein-based vaccine from GSK-Bio utilizes a combination of the TLR9 agonist CpG7909 and the TLR4 agonist MPL [36]. Other cytokines with immune-potentiating activities could be considered, including IL-12 [37-39]. Thus, while our study has identified an optimal dose of CEA peptide, further improvements in vaccine potency might be achievable through optimization of the adjuvant component.

In addition to utilizing vaccination to increase the frequency of tumor-reactive T cells, features of the tumor microenvironment can be dominantly suppressive and it may be necessary to inhibit such factors for optimal clinical activity. These include immunosuppressive cytokines such as IL-10 and TGF-β; expression of indoleamine-2,3-dioxygenase; the presence of myeloid-derived suppressor cells (MDSCs) and regulatory T cells (Tregs); inhibitory ligands such as B7-H1/PD-L1; and the dense tumor stroma that is characteristic of this disease [40,41]. Recently, using an agonist CD40 mAb in combination with gemcitabine, Beatty et al. demonstrated a partial reversal of immune suppression in PC and highlighted the role of macrophages in this process [42]. Future studies combining an optimized vaccine formulation along with manipulations to favorably alter the PC tumor microenvironment will be attractive to consider.

## Conclusions

We conducted a randomized phase I trial of carcinoembryonic antigen (CEA) peptide (CAP1-6D)/Montanide/GM-CSF-vaccine in 19 pancreatic cancer patients. Our primary objective was to find the optimal dose of the vaccine. The dose that induced a maximal T cell response was 1 mg and no significant toxicity was observed. Several long-term survivors with metastatic or locally advanced disease were noted. Future studies can build on these results by combining the above vaccine peptide dose with stronger adjuvants and/or agents to favorably alter the tumor microenvironment.

## Competing interests

Drs. Geynisman, Zha, Kunnavakkam, Aklilu, Catenacci, Polite, Rosenbaum, Namakydoust, Karrison, Gajewski and Kindler declare that they have no competing interests.

## Authors’ contributions

DG coordinated and analyzed the data as well as wrote the manuscript; YZ carried out the immunoassays and helped to analyze the data; RK helped with the statistical analysis; MA helped in the original design of the study; DC helped to conduct the study; BP participated in the design of the study and manuscript writing; CR helped to conduct the study; AN helped in the initial data analysis and manuscript preparation; TK helped with the initial statistical design of the trial; TG conceived of the study, participated in the design, immunologic analysis and writing of the final manuscript; HL conceived of the study, oversaw the study’s clinical implementation and participated in the design and writing of the final manuscript. All authors read and approved the final manuscript.
